# Development of a web-based patient decision aid for initiating disease modifying anti-rheumatic drugs using user-centred design methods

**DOI:** 10.1186/s12911-017-0433-5

**Published:** 2017-04-26

**Authors:** Ingrid Nota, Constance H. C. Drossaert, Heleen C. Melissant, Erik Taal, Harald E. Vonkeman, Cees J. Haagsma, Mart A. F. J. van de Laar

**Affiliations:** 1 Department of Psychology, Health and Technology, University of Twente, PO Box 217, 7500AE Enschede, The Netherlands; 2 Department of Clinical Psychology, VU University Amsterdam, Van der Boechorststraat 1, 1081 BT Amsterdam, The Netherlands; 3 Department of Rheumatology and Clinical Immunology, Medisch Spectrum Twente, PO Box 50 000, 7500 KA Enschede, The Netherlands; 4 Department of Rheumatology, Ziekenhuisgroep Twente, PO Box 7600, 7600 SZ Almelo, The Netherlands

**Keywords:** Patient Decision Aid, Shared Decision-Making, International Patient Decision Aids Standards, User-centred design

## Abstract

**Background:**

A main element of patient-centred care, Patient Decision Aids (PtDAs) facilitate shared decision-making (SDM). A recent update of the International Patient Decision Aids Standards (IPDAS) emphasised patient involvement during PtDA development, but omitted a methodology for doing so. This article reports on the value of user-centred design (UCD) methods for the development of a PtDA that aims to support inflammatory arthritis patients in their choice between disease modifying anti-rheumatic drugs (DMARDs).

**Methods:**

The IPDAS development process model in combination with UCD methods were applied. The process was overseen by an eight-member multidisciplinary steering group. Patients and health professionals were iteratively consulted. Qualitative in-depth interviews combined with rapid prototyping were conducted with patients to assess their needs for specific functionality, content and design of the PtDA. Group meetings with health professionals were organized to assess patients’ needs and to determine how the PtDA should be integrated into patient pathways. The current literature was reviewed to determine the clinical evidence to include in the PtDA. To evaluate usability among patients, they were observed using the PtDA while thinking aloud and then interviewed.

**Results:**

The combination of patient interviews with rapid prototyping revealed that patients wanted to compare multiple DMARDs both for their clinical aspects and implications for daily life. Health professionals mainly wanted to refer patients to a reliable, easily adjustable source of information about DMARDs. A web-based PtDA was constructed consisting of four parts: 1) general information about SDM, inflammatory arthritis and DMARDs; 2) an application to compare particular DMARDs; 3) value clarification exercises; and 4) a printed summary of patients’ notes, preferences, worries and questions that they could bring to discuss with their rheumatologist.

**Conclusions:**

The study demonstrated that UCD methods can be of great value for the development of PtDAs. The early, iterative involvement of patients and health professionals was helpful in developing a novel user-friendly PtDA that allowed patients to choose between DMARDs. The PtDA fits the values of all stakeholders and easily integrates with the patient pathway and daily workflow of health professionals. This collaborative designed PtDA may improve SDM and patient participation in arthritis care.

**Electronic supplementary material:**

The online version of this article (doi:10.1186/s12911-017-0433-5) contains supplementary material, which is available to authorized users.

## Background

Shared decision-making (SDM) is one of the main activities of patient-centred care [1, 2]. It involves the exchange of information and negotiation between the clinician and the patient to agree on the best way to medically proceed for the individual patient [3]. Often the decision-making process is complex - especially when preference sensitive aspects are involved. Various interventions have been developed to facilitate SDM.

Patient Decision Aids (PtDAs) are intended to support patients in making specific and deliberated choices among healthcare options [4–6]. In contrast to more general health education materials (e.g. information leaflets), PtDAs specifically state the decision being considered and stress the relevance of a SDM process [4–6]. Furthermore, PtDAs provide information on all available treatment options and help patients clarify what matters to them regarding these treatment options [4–6]. A systematic review recently revealed that, for many different decisions and conditions, PtDAs can improve patients’ knowledge about options, risk perceptions, feelings of being informed and being certain about what matters to them [7]. Furthermore, with the use of PtDAs, patients more often reach decisions that are consistent with their personal values [7]. Finally, PtDAs can improve patient-doctor communication [7].

The International Patient Decision Aids Standards (IPDAS) Collaboration states that the development of PtDAs should be systematic and include consultations of patients and health professionals [4–6]. However, many studies of PtDA development projects do not report on having involved patients during their development [8]. In response to this omission, the IPDAS’ evidence base has recently been updated to include a development process model that places more emphasis on patient involvement during PtDA development [6, 8]. This process model provides a step-wise approach to careful and systematic development, evaluation and implementation of PtDAs. Although this new comprehensive model provides an overview of the entire development process, it does not provide guidance on *how* to best involve patients and health professionals nor which research methods to use. The authors, therefore, urged PtDA developers to complement the IPDAS development process model with other guidelines, such as a user-centred design approach [8]. In a user-centred design, specific research methods are used to consult with potential users relatively early within the developmental timeframe [9, 10]. This approach allows developers to adopt and implement user-centred input, resulting in the product more adequately fulfilling users’ needs and, consequently, positively affecting user satisfaction [9, 10].

While patients with rheumatic diseases often face long-term treatment decisions, only a few studies have been reported on PtDAs for initiating disease modifying anti-rheumatic drug (DMARD) therapy [7, 11–13]. DMARDs are the core element of the management of inflammatory arthritis in order to control the disease process and to relieve or reverse symptoms [14–16]. DMARDs form two major classes: synthetic chemical compounds (sDMARDs) and biological agents (bDMARDs). With regard to DMARDs, the decision-making process has become increasingly complex, as numerous therapeutic options are available. In addition, new treatment strategies are rapidly evolving, but without sufficient information on differential efficacy and safety [14–16]. When weighing the options, elements to consider include treatment efficacy, approximate time-to-benefit, possible side effects, current and future risks, cost-effectiveness, route of administration and impact on daily life. Because this complex decision-making process concerns both clinical and preference-sensitive aspects, the choice of treatment needs to be based on a shared decision between the patient and rheumatologist.

The reported PtDAs for initiating DMARDs mainly focus on the decision whether to initiate one specific DMARD or a particular class of DMARDs [7, 11, 13]. Yet, our previous study showed that patients would like to be informed about multiple specific DMARDs [17]. In fact, previous research has shown that patients with a rheumatic disease are often less informed and less involved in decision-making than they would prefer [17–24]. SDM barriers reported by patients include being unaware of having a choice, lack of medical knowledge and a power imbalance in the doctor-patient relationship [17, 25].

In order to fulfil this need of patients with rheumatic disease, the aim of this study was to develop a tool that could compare multiple specific DMARDs. This paper describes the development of a web-based PtDA with use of the IPDAS development process model and user-centred design methods. The PtDA is intended for inflammatory arthritis patients who face the decision whether to initiate DMARDs.

## Methods

**Fig. 1:**
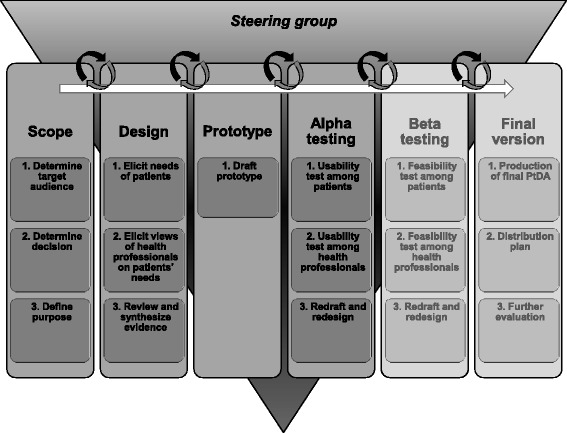
The IPDAS Development Process Model [8]

To develop the PtDA, the IPDAS development process model was used in combination with user-centred design methods. As illustrated in Fig. 1, the IPDAS process model provides a careful and systematic step-wise approach to develop PtDAs that are user-tested and open to scrutiny [8]. The current study focused on the first four steps of the IPDAS process model: ‘scope,’ ‘design,’ ‘prototype development’ and ‘alpha testing,’ which are further described below. The process was overseen by a multidisciplinary steering group, consisting of three rheumatologists (including one epidemiologist), a rheumatology specialist nurse, three experts in SDM and health psychology, and an experienced web designer.

### Scope

Because our previous studies showed a need for patient participation and information about multiple specific DMARDs [17, 24], the steering group decided to develop a PtDA for patients diagnosed with Rheumatoid Arthritis (RA), Ankylosing Spondylitis (AS) or Psoriatic Arthritis (PsA) who face the decision to initiate (a different) DMARD.

### Design

#### Needs assessment among patients (Design 1)

In-depth semi-structured face-to-face interviews were conducted (by IN) to assess patients’ needs for functionalities, content and design of the PtDA. We recruited 32 patients diagnosed with RA, AS or PsA, who recently (<1 month ago) consulted their rheumatologist and discussed initiating a (different) DMARD.

The interviews lasted between 45 and 120 minutes, were recorded and consisted of two parts. The first part of the interview explored what considerations, worries and questions patients had when deciding about DMARDs and what information patients needed in order to participate in the decision-making process. These findings helped determine what information the PtDA needed to provide. This part of the interview has been reported elsewhere [26].

The second part of the interview focused on patients’ needs regarding the functionalities, content and design of a PtDA for initiating DMARD therapy. To introduce the concept of a PtDA, we gave a general description. As a picture is worth a thousand words, rapid prototyping [27] was conducted to assess the usefulness of several potential PtDA features. A prototype was drafted with use of the software application Evolus Pencil 1.2 [28]. Previous developed PtDAs (e.g. [29, 30]) and the stepwise model for SDM developed by Elwyn and others [31] were used as an inspiration for the steps to guide patients through the decision-making process (i.e. acknowledging a decision needs to be made, gaining knowledge about options, preference eliciting and preparation for the decision talk).

The prototype included an innovative application to compare medications. This application was a direct response to findings from previous studies in which patients’ expressed the need for information about multiple options [17] and was inspired by commercial web-applications that allow consumers to compare various product features. The prototype was printed on paper and appears in Additional file 1 (in Dutch). Each page in Additional file 1 is a copy of the 10 prototype screens in the same order in which they would appear online.^1^

The interviewer and patients walked through the paper prototype and discussed usability issues and additional needs (regarding functionalities, content as well as design). Patients’ remarks were written on the paper prototype and later analysed alongside the analysis of the audio recordings.

Interviews were transcribed verbatim and analysed using Atlas.ti 7.1 [32], a qualitative analysis software application which allows researchers to overview the codes, link statements and visualize connections between themes [33]. Furthermore, this software can also integrate pictures - in this case, the paper prototype with written remarks of the participants. The analysts (IN, CHCD and HCM) mutually independently analysed the data using the principle of constant comparison [34] and an iterative process of deductive and inductive analysis. First, all quotes were (deductively) categorized into needs for functionalities, content and design. These quotes were then further analysed using a process of open coding (inductive analysis), followed by axial and selective coding (deductive analysis) [34, 35]. During this process the analysts preserved the voice of the patients. After each phase, the individual findings were compared and analysed until consensus was reached. Finally, in close collaboration with the web designer, the analysts translated the needs (in the voice of the patients) into a list with requirements.

#### Needs assessment among health professionals (Design 2)

In accordance with user-centred design theories, all stakeholders need to be consulted during development [9, 10]. To comply with this requirement, all rheumatologists (*n* = 11), specialized nurses (*n* = 3) and rheumatology nurses (*n* = 4) of the two participating hospitals were invited to participate in a semi-structured group interview. They were asked to give their expert opinion on functionalities, content, design and distribution of a PtDA for initiating DMARD therapy.

Firstly, health professionals were asked to indicate what information patients needed to know before being able to make a decision and how this information should be presented. Then, the paper prototype was presented and participants were asked to express their expert opinion regarding its functionalities, content and design. After that, the results of the needs assessment among patients were presented, and participating health professionals were asked to reflect on them.

Secondly, to be able to determine how to distribute the PtDA and how to best integrate the PtDA into clinical practice, the health professionals were asked to outline the patient pathway and to discuss when and how to refer patients to the PtDA (setting and timing). The patient pathway was outlined by following the steps of regular patients from their appointment to discuss initiating DMARDs with the rheumatologist to taking their first DMARD dosage. Patient pathways were outlined for newly diagnosed patients as well as for patients with a longer history of RA, AP or PsA.

During the meeting, we explored the range of opinions and aimed for consensus on the health professionals’ needs for the PtDA. Notes were taken by two members of the steering group (IN and HCM). Similar to the analysis of the needs assessment among patients, the analysts (IN and HCM) mutually independently analysed the notes using the principle of constant comparison [34] and a combination of deductive and inductive analysis. The notes were first classified into the needs for functionalities, content, design and distribution/implementation (deductive analysis). Then, the notes were inductively analysed with a process of open coding, which resulted in the categories arising from the data. The coding ended with deductive analyses (axial coding and selective coding) [34, 35]. After each phase, the findings of the analysts were compared and further analysed and discussed until consensus was reached. Then, the needs (in the voice of the health professionals) were translated into a list of PtDA requirements for content, design and distribution/implementation. To confirm whether we translated the needs correctly, the list was sent by email to the health professionals, all subsequently agreed on the listed items.

#### Evidence review (Design 3)

The background information and clinical evidence included in the PtDA were based on the needs assessments among patients and health professionals, availability and quality of the evidence. We reviewed current international guidelines on the management of RA, AS and PsA [14–16] which provide recommendations on general aspects of treatment, mostly on group drug levels (e.g. sDMARDs and bDMARDs). Furthermore, we reviewed medication information leaflets from the participating hospitals, the local pharmacists, the Dutch Arthritis Association [36], the “Farmacotherapeutisch Kompas” (a Dutch database that encompasses independent information on all drugs available in The Netherlands) [37], and the information pharmaceutical companies provide to health professionals and patients.

### Working prototype

Based upon the needs assessments with the various stakeholders (patients, health professionals and the steering group), the IPDAS criteria [5, 6] and the evidence review, the paper prototype of the PtDA was redrafted and redesigned. First, the steering group developed a plan for integrating the PtDA into the patient pathway, then the PtDA was redesigned and programmed into a working prototype. Additional file 2 shows the redrafting process of the PtDA screen that enabled patients to compare DMARDs.

### Alpha testing

#### Usability test among patients (Alpha testing 1)

A usability study was conducted with the working prototype of the PtDA. Patients with RA, PsA, or SA were recruited from the “Patient Research Partners”-Panel of the Arthritis Centre Twente and via the two participating hospitals.

Data were collected by observing the patients’ usage of the PtDA and semi-structured interviews conducted during and after usage by two of the authors (IN and HCM). First, participants were asked about their demographics, their perceived health status on a scale ranging from 1 indicating ‘poor health’ to 10 for ‘excellent health,’ their history with regard to decision-making about DMARDs and their experiences with online health information. They were then presented a scenario describing a possible decision (closely matching their history with decision-making about DMARDs) and a brief description of the rheumatologist’s consultation that referred them to the PtDA. They were subsequently given a referral card containing the internet address and the treatment options suggested by the rheumatologist and were assigned to visit the PtDA website. While using the PtDA, participants were asked to think aloud [38, 39]. When visiting the homepage, they were interrupted briefly and questioned about their expectations of the website. After observation of their free usage of the PtDA, the semi-structured interview started which included questions about *perceived usefulness, perceived ease of use, attitude towards using* and *intention to use*. These elements are based on the Technology Acceptance Model (TAM) [40, 41]. The sessions lasted between 45 and 97 minutes.

We used Morae 3.3.0 [42], a software application for usability testing, to record the performance task and the interview. This programme records a video of the user (including sound), screen activity and system events (including mouse clicks, web page changes and onscreen text).

After completing all sessions, two analysts (IN and HCM) selected relevant written remarks of participants, watched the videos of the usage and interviews and made notes on their observations.. The analysis mainly focused on the correspondence between the structure of the website and the cognitive steps the users followed. The notes were linked to specific pages of the website (e.g. the homepage or the page enabling comparison of medication) and to the topic of discussion (e.g. structure, navigation, content, format, and colour). This resulted in a list of positive remarks as well as of points for generally improving the PtDA and each screen in particular.

#### Usability test among health professionals (Alpha testing 2)

To evaluate usability from the health professionals’ perspective, all rheumatologists (*n* = 11), specialized nurses (*n* = 3) and rheumatology nurses (*n* = 4) of the two participating hospitals were invited to participate in another semi-structured group interview. The aim of this usability study was focused on acceptability and compatibility of the PtDA and the current process of medical decision-making. This approach was taken since health professionals would not directly be using the website, but instead referring patients to the website. Health professionals would then need to interact with patients once they had used the website.

During the meeting, the working prototype of the PtDA was presented (by IN) and the health professionals were asked to give their opinion on the content and design of every screen. Notes were taken by a member of the steering group (HCM). After the presentation, the group was asked to test the working prototype individually, write down their remarks and reflect on *perceived usefulness, perceived ease of use, intention to use* and *compatibility* with the current process of medical decision-making. These elements are based on the Technology Acceptance Model (TAM) [40, 41].

Two analysts (IN and HCM) mutually independently read all the notes and, using inductive analysis, coded relevant remarks of the participants. The analysis mainly focused on compatibility with the current process of medical decision-making and intention to use. This resulted in a list of positive remarks as well as elements that could be improved.

#### Redraft and redesign of PtDA (Alpha testing 3)

Based on the results of the usability tests, an iterative draft-review-revise process by the steering group was conducted until the PtDA reached content and format ‘saturation’ (i.e. all points for improvement were accounted for). Overall, no major adjustments were conducted, and hence the steering group decided to forego another usability study.

## Results

### Design

#### Needs assessment among patients (Design 1)

In total 26 women and 6 men participated, with an average age of 54 years. Most participants (62%) had completed 12–16 years of education and were currently employed (56%). Some participants (*n* = 5) had discussed initiating their first DMARD with their rheumatologist. Others (*n* = 27) were already using sDMARDs or bDMARDS and had discussed changing to another DMARD with their rheumatologist.

The first part of the interview aimed at deepening our understanding of patients’ considerations when deciding about DMARDs and what information patients need to participate in the decision-making process. The results of this part of the interview have been published elsewhere [26]; but briefly, patients felt the need for a complete overview of treatment options. Results also showed that before deciding about DMARDs, arthritis patients wanted information with regard to both clinical features (e.g. aim and working mechanism, time-to-benefit, manner of administration, potential side effects and risks, influence on fertility and pregnancy) as well as possible consequences for their daily lives (e.g. restrictions on driving a vehicle and alcohol consumption and how to fit the treatment schedule into their daily lives). Finally, patients mentioned many concerns and questions that could be incorporated into the value clarification exercises (i.e. the lists of common worries and questions).

The second part of the interview introduced the concept of a PtDA. In general, participants were positive about implementing a PtDA for choosing between DMARDs. In line with our previous research [17, 24], a few participants did not feel the need for a PtDA, because they did not want to participate in medical decision-making or because they felt the current information was sufficient. For example: *“I do find that easy, to just leave [the decisions] behind at the doctor’s”* and *“The information given by the rheumatologist is good enough for me.”* Most participants liked the idea because it would be a reliable source of information, to help them prepare for the decision-making consultation. This is illustrated by the following quotations:

*“This way you can keep [all the information] together, without having to look for it. And when your doctor refers you to it, well, then it has to be trustworthy.”*

*“In this way you can really prepare yourself for the decision-making consultation, having some idea beforehand of what to expect. If you go to the rheumatologist without having the slightest notion, then, after some time, there still are questions you might not have asked.”*

Furthermore, a PtDA would fit patients’ need to be informed about multiple treatment options for their present situation as well as for the future. To quote one participant:

*“When you consult your doctor, it is only a ten-minute-conversation, ending with a prescription that you think is alright – and when it actually helps, it is alright indeed. Yet there are perhaps many other possibilities, with less side-effects or smaller doses …, and the doctor will certainly not explain all of them … This tool enables us to have insights into all the possibilities, to work in a structural way and say, ‘All right, this is where you are, and from here you can go either in this or in that direction … And when this does not work, you can go in that other direction.”*

Reviewing the paper prototype, most participants liked that the PtDA provided insights into all available medication options. However, some had their doubts. In The Netherlands, therapy with a biologic DMARD is reimbursed for patients with at least moderate disease activity for whom treatment with at least two synthetic DMARDs has failed. Some participants felt that it could be frustrating to receive information about medication for which they were not (yet) eligible: *“It is frustrating indeed when something is not available, because [the insurance company] considers it to be too expensive.”* Some of them suggested to solve this by tailoring the appropriate options to the individual patient.

Almost all participants liked the opportunity to compare treatment options for different features in a structured manner prior to the decision, as illustrated by the following quotation:

*“In this way you get a clearer idea about different kinds of medicines. Normally you receive only information about the drug you start with, like, ‘This is what you have to take, so there you go’. This does not leave you with a clear idea about whether another medicine in the same category is perhaps more suitable. I think that this other approach does help to sort it all out a little better.”*

Participants also appreciated that the prototype provided, besides clinical benefits and risks, practical information with possible implications for patients’ daily lives. They suggested a variety of categories that should be added in the side-by-side comparison, including: restrictions for nutrition and alcohol, storage instructions, influence on daily routine and guidelines for traveling. Most participants did not value the personal stories of peers that were included for each DMARD because *“every patient is different,” “they will probably be actors”* or *“that is not reliable information and does not belong on such a website.”*

All participants liked that the information was provided in portions; the paper prototype suggested that supplemental information could be obtained via links that would unfold elements of the webpage. Some of the information in the paper prototype was provided using pictograms, pictures or videos. Although some pictograms needed clarification, many respondents asked if we could add more pictograms and decrease the amount of text. To further reduce the amount of text, some participants suggested tailoring the content of the information (e.g. risks) to the individual patient.

The pictures and videos illustrating the administration of the treatments were appreciated by most respondents. Such illustrations can decrease uncertainty and anxiety, especially when the medication requires the administration of injections or infusions. To quote two participants:

*“It really was a relief to see that injection needle, which was quite different from what I expected. So I believe that when people see such short instructive films, they can be better prepared for [their treatment].”*

*“If you watch [such a short film], you know beforehand what you are getting yourself into.”*

With regard to the proposed value clarification exercises, most thought this would be helpful to their decision-making process. Some participants mentioned that thinking about their preferences would support them in participating in decision-making. One participant hypothesized that *“this may increase patients’ feelings of being in control”*. Many participants also appreciated the lists of worries and questions. They felt that the lists acknowledged that it is normal to have these worries and questions and thought that it would support them to express these feelings and questions during their next consultation with the rheumatologist. For example, one participant said: *“The question I had is one of those addressed here [on the list], so it doesn’t seem to be such a strange question, but one I can ask without any fear.”*

Participants also expressed that they would like to bring the summary containing their notes, preferences, worries and questions to their consultation as a reminder and to increase their confidence in their ability to participate in medical decision-making. To quote one participant: *“Everything is really more focused, like, ‘I see you have prepared yourself and that you still have the following questions, so let us start with those’.”*

Most participants expressed that they intended to use the PtDA, especially if it was available at home which would allow them to use it at their ease. A few mentioned that they would like to use the PtDA, but were afraid it would take too much time and effort to go through all the steps. Finally, a couple of participants did not like the PtDA to be computer-based, because they felt that they lacked sufficient computer skills or did not have access to a computer/internet.

The needs elicited from this study were translated into requirements of the PtDA, as presented in Appendix 1.

#### Needs assessment among health professionals (Design 2)

Ten rheumatologists, two specialized nurses and two rheumatology nurses were present at the group meeting. Most health professionals were eager to implement a PtDA into their practice, considering it an innovative way to inform patients. Their primary reason for adoption was to be able to refer patients to a website with reliable health information. However, some health professionals were sceptical at first. They thought the PtDA would be time-consuming without adding value to the current information leaflets from the hospital. However, when they learned (from the results of the needs assessment among patients) that many patients desired more information than they currently received and that they would like to be able to compare DMARD options with regard to clinical elements as well as possible consequences for their daily lives, the health professionals understood the added value of a PtDA.

The health professionals discussed when and how to refer patients to the PtDA by outlining patients’ pathways. All of the health professionals worried that patients might become overwhelmed when informed about all available DMARDs. Therefore, all agreed that patients should be referred to the PtDA only after having consulted their rheumatologist or specialized nurse who would first provide them with a personal recommendation for appropriate options for medication. The health professionals disagreed on whether patients should be able to see all options for medication or only the ones that were personally recommended. After some discussion, consensus was reached; patients should receive a clear personal recommendation in writing (preferably digital), but should be free to also read information about other DMARD options in the PtDA. The health professionals were asked if they had specific requirements for the PtDA. Some mentioned they would like to read patients’ preferences, worries and questions before the encounter, but others felt that this would be a violation of privacy and that it was the patients’ right to decide what to share with their health professional. Another requirement of the health professionals was to be able to easily add new drugs to the PtDA because of the rapid development of new DMARDs. Appendix 2 presents an overview of the requirements based on the needs assessment among health professionals.

With regard to the needs of the steering group, one additional item was included in the list of PtDA’s requirements. For the purpose of evaluating the usage of the PtDA, the steering group wanted the website of the PtDA to log anonymous user data (navigation and input).

#### Evidence review (Design 3)

When reviewing the clinical evidence, we discovered there was insufficient evidence on differential efficacy and safety. Therefore, it was difficult to use the available clinical information for detailed comparisons of DMARDs. Not only was information unequally available for each drug, but it was also conflicting. In such cases we based the information in our PtDA on consensus between the rheumatologists of the participating hospitals (*n* = 11).

Two informal group meetings with all rheumatologists, a specialized nurse and three members of the steering group (IN, HV and ML) were organized to discuss the clinical evidence. Taking into account the information needs of the patients, the rheumatologists decided, in general, what information: 1) must be disclosed to all patients, 2) should be provided as supplemental information for patients who desire additional information, and 3) need not be included at all. With this information, we were able to develop a flexible information system which would fulfil the needs of most users without overwhelming others. During the second meeting, the unclear and conflicting information was presented and discussed until consensus was reached among all rheumatologists. The final texts were checked by five rheumatologists.

### Working prototype

**Fig. 2:**
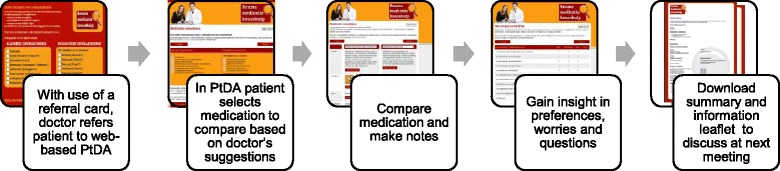
Process of the Patient Decision Aid

Based upon the results of the previous studies and the evidence review, we developed a working prototype and a plan for integration of the PtDA into the patient pathway (illustrated in Fig. 2). According to this plan, the patient and rheumatologist have an initial conversation about starting a (different) DMARD. During this conversation, the rheumatologist refers the patient to the web-based PtDA along with a referral card. On this referral card, the rheumatologist indicates the DMARDs appropriate for the patient at this specific moment. The referral card states the internet address of the PtDA, and the patient is encouraged to access the PtDA at home.

The working prototype of the website consisted of two components: general information and the PtDA itself. The component with general information addressed SDM, emphasized the importance of the patient’s role in medical decision-making, and provided general information about inflammatory arthritis and DMARDs. The component with the PtDA (see Fig. 2) consisted of an application to compare selected DMARDs side-by-side. Elements that were compared included: target and working mechanism of the medication, manner of administration, approximate time-to-benefit, risks of side effects, follow-up process, combination with other drugs, fertility/pregnancy, consequences of continuing or stopping the DMARD, drug marketing history, restrictions and warnings for nutrition and alcohol consumption, and impact on daily life (e.g. storage, daily routine, traveling). In order to fulfil the high need for information of most users while not overwhelming others, the PtDA has a flexible information system - supplemental information about the medication can be obtained via links that unfold certain elements. Furthermore, the working prototype included a digital notebook, which included exercises to gain insight into one’s own preferences, worries and questions along with a summary that compiled the patient’s exercise responses. This summary could be downloaded, printed and later used during the patient’s next consultation with the rheumatologist.

Some wished-for attributes of the PtDA were not realized due to their technical complexity, time and financial limitations, and/or privacy issues (see Appendices 1 and 2). The steering group weighed the needs of both patients and health professionals. For example, many patients expressed the need to have an overview of all available DMARDs, while others only want information about medication that is personally recommended to them. In order to not overwhelm patients, health professionals concluded that patients should receive a clear personal treatment recommendation in writing -preferably digital-, but should be free to also read information about other DMARD options in the PtDA. A solution would be to tailor the PtDA to the information needs of each patient. This would require patients to register online before accessing the PtDA. However, this solution raises privacy concerns and would withhold some patients to use the PtDA. Therefore the steering group decided to use the referral card – It provides patients with an overview of treatment options combined with a clear recommendation from the rheumatologist. When patients access the web-based PtDA, they are asked to select the medication that was recommended by their rheumatologists, but the information on other available medication options is freely accessible.

Tailoring the information to the patients individual risk profile proved too time consuming and out of budget. Also, sending the summary of the patient’s notes, preferences, worries and questions directly to the health professional was not implemented because of privacy concerns.

### Alpha Testing

#### Usability test among patients (Alpha testing 1)

A total of 5 women and 5 men participated in the usability study, with an average age of 55 years (range 31–85 years). The participants were heterogeneous with regard to their educational status and current employment (1 participant finished primary education, 4 achieved intermediate education and 5 achieved higher education; 6 participants were employed). They were also heterogeneous regarding their disease-related internet use. On average, the participants each spent 11 hours per week online (range 2–35 hours per week). All had at least searched once online for information about arthritis, treatments and health services. Most (*n* = 8) had ordered their medication online, but only a few (*n* = 2) had used interactive health websites (e.g. online consultation).

All participants were diagnosed with RA. At the moment of the usability test, 4 participants experienced a poor health status (score <5). Some respondents (*n* = 4) had been diagnosed in the past year and had only discussed initiating DMARDs once or twice (only sDMARDs). Others (*n* = 6) had a longer disease duration and had previously decided to initiate sDMARDs as well as bDMARDs.

When visiting the homepage, most participants mentioned the working prototype had a clear structure and professional appearance. When asked about their expectations, some expected the PtDA to result in a treatment recommendation, but most correctly expected the PtDA to give them the opportunity to compare DMARDs.

During our observations of patients’ free usage and walkthrough of the working prototype, we discovered some significant barriers to usability. Firstly, we discovered that the referral card was not easy to use; the card was printed on both sides, one side for the sDMARDs and the other for the bDMARDs. Not all participants noticed this and, therefore, did not have an overview of all DMARDs supposedly ticked by the rheumatologist. Secondly, we discovered early in the study (after three observations) that participants had difficulties navigating the screen that allowed them to compare DMARDs. All three participants did not know what to do, stopped and asked for help. We asked the three participants for tips to improve navigation, and, based on their suggestions, we made a paper prototype of the revised screen. We added this paper prototype during the usability test with subsequent participants (*n* = 7). Most of them liked the revised page and thought it would be easier to use. Thirdly, we observed that after completing the comparison of DMARDs, some participants felt they finished the PtDA. They were not aware that more steps followed. This was because the button for the next step was not prominently visible. We asked participants to suggest a better location for the button and how to highlight the next steps of the PtDA. Finally, a few small programming errors were found.

The interviews largely confirmed the results of the observations; overall, participants mentioned that the working prototype was easy to use and the information easy to read. The PtDA was perceived as useful with regard to comparing DMARDs side-by-side; gaining insights into preferences, worries and questions; and having all the information on one reliable location, all of which might support their decision-making process, as illustrated by the following quotations:

*“The tool to compare medication is very useful – every aspect [of the medication] can be compared side by side.”*

*“They < the value clarification exercises > help you prepare < for the next consultation>. I think I would be able to ask better questions and I would feel less insecure.”*

*“All information is in one place, a reliable source, so you do not have to search anymore.”*

*“This helps me to think systematically about my options.”*

Furthermore, many participants appreciated the pictures and videos visualizing the manner of administrating the medications. Most suggestions were directed at clarifying the PtDA steps and improving the navigation on the screen that enabled the comparison of DMARDs. Minor remarks included clarifying some specific content and decreasing the amount of text. With regard to intention to use, most participants said that they would use it, some would not, but all would recommend it to others.

#### Usability test among health professionals (Alpha testing 2)

Nine rheumatologists, one specialized nurse and two rheumatology nurses attended the group meeting. Overall, all health professionals appreciated the clear structure of the website and the clarity of the text. Similar to the results of the usability test among patients, some health professionals perceived the navigation on the screen that enabled comparison of DMARDs to be rather complex. Other screens were perceived as easy to use. Most health professionals believed that the PtDA would be very useful, especially to gain insight into patients’ preferences, worries and questions and to discuss these topics with them. A few remained sceptical about the added value (see our previous discussion of the needs assessment among health professionals), but were willing to try using it. All health professionals thought the PtDA would be highly compatible with the regular process of medical decision-making and easily implemented.

#### Redraft and redesign of PtDA (Alpha testing 3)

Based on the results of the usability tests, the steering group discussed which adjustments needed to be implemented. The referral card was adjusted to print on one side only, allowing a clear overview of all treatment options and the personal recommendation of the rheumatologist for appropriate medication. To highlight the steps of the PtDA, we altered some of the text on the website and added an instructional video (see Additional file 4). The navigation on the screen that enabled comparison of DMARDs was adjusted according to the recommendations of the participants. We did not increase the amount of medications to compare side by side because the font would then become too little to read. Some buttons were relocated, and several programming errors were fixed. Overall, no major adjustments were necessary. Additional file 2 shows how the page that enabled comparison of DMARDs was redrafted from paper prototype to working prototype and the final version. The card rheumatologists use to refer patients to the PtDA can be found in Additional file 3. Additional file 4 contains the instructional video of the PtDA and provides a clear representation of the PtDA and its functionalities.


Additional file 4: Video S1. Instructional video of the Patient Decision Aid. Description: Instructional video of the Patient Decision Aid (in Dutch). (MOV 11220 kb)


## Discussion

We have described in detail the development of a PtDA for patients with inflammatory arthritis that helps them to choose between DMARDs. This PtDA was developed using the IPDAS development process model [8] and user-centred design methods [9, 10]. Based upon the needs assessments of both patients and health professionals, we constructed a web-based PtDA consisting of the following parts: 1) general information about SDM, inflammatory arthritis and DMARDs; 2) an application to compare specific DMARDs attributes; 3) exercises to gain insight into the patient’s preferences, worries and questions; and 4) a printed summary of the patient’s notes, preferences, worries and questions to be discussed with the rheumatologist at the next consultation. The results of the alpha tests revealed that the developed PtDA largely satisfied the needs of both patients and health professionals and thus has the potential of being a valuable tool for patients who need to choose between DMARDs.

The overall process of development was satisfactory. The IPDAS development process model is relatively new and has yet to be substantially tested. Nevertheless, this process model proved to be systematic and helpful to our iterative development of the PtDA as well as compatible with user-centred design methods. In addition, the user-centred design methods proved to be helpful in gaining valuable insights into different stakeholders’ needs with regard to the PtDAs content and design and how it should be integrated into daily practice.

Firstly, rapid prototyping (i.e. the use of paper prototypes) proved to be of additional value to the needs assessment interviews. Patients (but also clinicians) often have difficulty conceptualizing what a PtDA is and how it should look and function, which might limit them in expressing their needs. With the use of rapid prototyping, it was easier for users to express their wishes and needs and to give critical input. For this reason, we recommend using rapid prototyping in the development process of future PtDAs.

Secondly, according to the IPDAS development process model, health professionals’ perceptions of patients’ needs for information and decision support should be assessed. We recommend conducting this step after having elucidated the patients’ needs. In our study we intentionally conducted the study first among patients and presented the results of this study during the session with the health professionals. By doing so, the results of the needs assessments among patients were largely confirmed. But perhaps more essentially, this procedure proved to be effective in creating support among more sceptical health professionals for the development of the PtDA. Health professionals who initially questioned the added value of a PtDA had less misgivings and were more willing to use it.

Thirdly, we recommend not only asking health professionals about their perception of patients’ needs, but also asking them about their own needs and thoughts on implementing a PtDA into their practice. Their practical and expert knowledge on the decision-making process can be of great value for the integration of a PtDA into the patient pathway and daily workflow of health professionals, and consequently enhance the adoption and implementation of the PtDA. The adoption and implementation of PtDAs using a referral model (i.e. health professionals inviting eligible patients to use the PtDA) is often challenged by indifference on the part of health professionals [43]. This indifference may stem from a lack of confidence in the content of the PtDAs and concerns about disruption of established workflows [43]. Our PtDA is still being successfully used after conclusion of the project (after beta testing), and newly developed DMARDs have since been added by the health professionals. This indicates that the iterative and extensive involvement of health professionals and the acknowledgement of their needs for the PtDA were important in creating ownership.

Finally, as the scope of the internet grows, PtDAs will be more and more computerized and web-based. These formats may offer many opportunities, not only for rapid adjustments of the PtDAs, but also for studying usage and usage behaviour in detail. For instance, the amount of log-ins, page-views, and time spent on the PtDA could be logged, but also patterns of usage (e.g. How do users navigate? Which elements and combination of elements are often used? When do users drop-out?) and users’ input (e.g. selected preferences, worries and questions) [44, 45]. This information could be used to gain more insight into users’ (evolving) needs and improve the PtDAs usage, usability and impact. Therefore, we recommend adding researchers to the stakeholders of web-based PtDAs and advising researchers to include logging anonymous user data as a requirement for the PtDA.

Compared to most previously reported PtDAs, the PtDA in this study encompasses many treatment options [46]. Although patients have the right to be informed about all treatment options [47] and one of the quality domains of the IPDAS is to provide all options to patients [5, 6], we had a valuable discussion with the health professionals about whether to give patients access to all available medication options. Our previous studies [17, 26] showed that patients not only worry about the side effects and potential risks of their current or proposed treatment, but also had significant worries about the risks of future treatments and about ‘running out of options,’ should the proposed medication fail to work. To decrease this uncertainty, patients expressed a need to have an overview of all available options, for the time being as well as for the future. However, patients will most certainly be overwhelmed by all the different options and their pros and cons. To guide patients through this plethora of options, we chose to provide them in writing (the referral card) a clear personal recommendation of their most appropriate medication options. To respect their needs and rights, we also provided an overview of all other medication options and gave patients access to this information as well. This format may also be suitable for PtDAs that address multiple treatment options for other conditions, such as asthma or diabetes.

Previously developed PtDAs for initiating DMARDs mainly differ in the amount of treatment options that are included and how it is integrated in the patient pathway and distributed to patients. Most of these PtDAs focus on the decision whether to initiate one specific DMARD or a particular class of DMARDs, are to be used outside the clinical encounter and the plan for distribution is often unclear [7, 11, 13]. Only one other PtDA includes all DMARD options and a clear plan for integration in daily clinical care. It consists of a card deck to be used during the medical encounter and is developed for patients with limited health literacy or limited English language proficiency [12].

One limitation of the currently developed PtDA is that it does not present outcome probabilities. This is due to the large number of treatment options included and the lack of evidence on differential efficacy and safety. Presenting outcome probabilities is a quality domain of the IPDAS [5, 6]. Not presenting outcome probabilities limits the comparison of treatment options. However, the PtDA does present the negative and positive features in equal detail and a structured manner of the available treatment options for clinical and practical elements as well as the possible implications for daily life. This way of presenting outcomes was regarded as useful by the patients.

Furthermore, it should be noted that some wished-for attributes of the PtDA were not realized due to their technical complexity, limited time and finances, and privacy issues. A few (elderly) patients stated they did not want a computerized version of the PtDA because they feared that they lacked sufficient computer skills or did not have access to a computer/internet. Since it was only a few patients who stated this and because of limited time and finances, we did not develop a paper and pencil PtDA. However, we chose to acknowledge this need by having a computer available for patient use in the hospital and having a nurse guide the patient through the PtDA decision-making process.

The content of the PtDA is now tailored to the individual based on gender and desire to have children, but not on risk profile. With the insufficient evidence on differential efficacy and safety of DMARDs, this attribute remains a challenge for future research.

Due to time and financial limitations, it was also infeasible to develop a digital referral to the PtDA accompanied by a personal recommendation for appropriate medication options. Nevertheless, it is technically interesting to digitalize this process and may even improve the uptake. Sending the summary with the patients’ notes, preferences, worries and questions directly to the health professional was also not realized because it raised privacy issues. Perhaps in the future, the PtDA could incorporate an option that would allow patients to send their summary to their health professional. Such additional attributes might also help increase the users’ uptake.

Compared to the majority of PtDA developments, our PtDA substantially attempted to include all stakeholders. However, only small groups of participants were involved and all (patients and health professionals) were recruited from two hospitals. Although the number of participants in all our steps actually match the recommended numbers (see [27, 48, 49]), our results may not be generalizable. When further developing or distributing this PtDA, attention should be paid to involve a larger and wider group of users.

Additionally, we have not compared and assessed our development of a web-based PtDA using the IPDAS process model and user-centred design methods with the development a web-based PtDA in a different way. To assess this, two web-based PtDAs need to be developed using different methods, but using the same ideas or content as the starting point. In our view, this seems unfeasible and undesirable. From our study, however, we can say that our methodology did allow us to clarify needs and we were able to adapt the PtDA to these needs.

Finally, in this paper we have not addressed evaluating the effectiveness or impact of the PtDA. To evaluate whether the PtDA is successful in improving patient participation and supporting SDM, we have conducted a post-test only study with a historical comparison group (beta testing). The results are published elsewhere [https://arthritis-research.biomedcentral.com/articles/10.1186/s13075-016-1138-3]. To assess the use, appreciation and perceived impact from an expert perspective, we have recently conducted a focus group study with health professionals. These results are also being analysed at this moment.

## Conclusion

By combining the IPDAS development process model with user-centred design methods, patients and health professionals contributed to the development of a novel web-based PtDA. This PtDA aims to support arthritis patients in their choice between DMARDs after they have received suggestions for appropriate treatment options from their rheumatologist. We have successfully demonstrated that user-centred design methods were helpful in developing a user-friendly application and creating support for the adoption of the PtDA. With use of these methods, the PtDA fits the values of all stakeholders and easily integrates with the patient pathway and daily workflow of health professionals. It is our expectation that this design approach may ease the uptake of PtDAs.

## Additional files

Additional file 1: All paper prototypes used in the needs assessment. Description: Images of all the paper prototypes used to assess needs. (PDF 928 kb)

Additional file 2: Design process from paper prototype to working prototype and final version. Description: Screenshots of the page that allows patients to compare DMARDs, illustrating the design process from paper prototype to working prototype and final version. (DOCX 573 kb)

Additional file 3: Card to refer to the Patient Decision Aid. Description: Image of the card rheumatologists use to refer patients to the Patient Decision Aid. (DOCX 139 kb)

## Appendix 1

**Table 1:** Requirements of PtDA based on patients’ needs

Functional requirements
• PtDA encourages patients to participate in medical decision-making.
• PtDA provides overview of (all) available options for medication.
• PtDA provides the opportunity to compare options for medication.
• PtDA supports patients to gain insight into their preferences, worries and questions regarding medication.
• PtDA urges patients to express their preferences, worries and questions about initiating medication.
Requirements for content, design and distribution
• The PtDA includes information about the decision-making process, SDM and the importance of the role of the patient.
• Available options for medication are listed to provide an overview.
• Medication can be compared for:
o Clinical aspects: aim and working mechanism of medication, manner of administration, time to benefit, risks for side effects, follow-up process, combination with other drugs, influence on fertility/pregnancy, continuing or stopping medication and history of medication.
o Possible implications for daily life: restrictions for nutrition and alcohol, storage instructions, influence on daily routine and guidelines for traveling.
• Tailoring:
o Appropriate treatment options are tailored to individual patient.
o Content of information is tailored to individual patient based on gender.
o Content of information is tailored to individual patient based on desire to have children.
o Content of information is tailored to individual patient based on risk profile^a^.
• Information in PtDA is easy to read:
o Pictograms are used as much as possible to decrease amount of text.
o Pictures and videos are used to provide insight into administration of medication.
o Information is written in plain language with links to definitions.
o Information is provided in portions; amount and complexity of information can be adapted to individual needs.
• PtDA provides the opportunity to give value to specific treatment options and features of the specific treatment options.
• PtDA includes exercises to gain insight into patients’ preferences, worries and questions.
• PtDA provides a summary of patients’ notes, preferences, worries and questions which can be saved and printed.
• Can be used at home (i.e. outside the hospital).
• Does not take more than 30 minutes to complete (on average).

^a^Not realized in final PtDA

*PtDA* Patient Decision Aid*, SDM* Shared Decision-Making

## Appendix 2

**Table 2:** Requirements of PtDA based on health professionals’ needs

Functional requirements
• PtDA provides health information on inflammatory arthritis and DMARDs.
• PtDA provides health professionals insight into patients’ preferences, worries and questions about DMARDs.
Requirements for content, design and distribution
• The provided information is based on the highest level of evidence available.
• The PtDA is an extension of existing information patients receive (added value).
• The PtDA meets the criteria set by laws regulating patient education and informed consent.
• Referral to the PtDA is accompanied by a clear personal recommendation for appropriate options .for medication in writing
… preferably digital^a^
• Information about DMARDs other than those personally recommended by the rheumatologist to the patient is freely available.
• The PtDA provides patients the opportunity to share their preferences, worries and questions about DMARDs with their health professionals.
… by digitally sending these insights to the health professional before the consultation.^a^
• The PtDA easily integrates with the existing daily work process/patient pathway.
• The PtDA is not time-consuming for health professionals.
• The PtDA is easily adjustable for newly developed DMARDs.

^a^Not realized in final PtDA

*PtDA* Patient Decision Aid*, DMARD* Disease Modifying Anti-Rheumatic Drug

## Abbreviations

AS: Ankylosing Spondylitis
bDMARD: Biologic disease modifying anti-rheumatic drug
DMARD: Disease modifying anti-rheumatic drug
IPDAS: International Patient Decision Aids Standards
PsA: Psoriatic Arthritis
PtDA: Patient Decision Aid
RA: Rheumatoid Arthritis
SDM: Shared Decision Making
sDMARD: Synthetic disease modifying anti-rheumatic drug

## Footnotes

1 The term ‘page’ is used to indicate a page of the paper prototype, whereas ‘screen’ indicates a screen on the website.
