# Supplementary material for: Development of a web-based patient decision aid for initiating disease modifying anti-rheumatic drugs using user-centred design methods
Source: BMC Med Inform Decis Mak. 2017 Apr 26;17:51. doi: 10.1186/s12911-017-0433-5 (PMC5405550; doi:10.1186/s12911-017-0433-5)
Supplement: Supplementary file 2 — All paper prototypes used in the needs assessment. Description: Images of all the paper prototypes used to assess needs. (PDF 928 kb) [file 12911_2017_433_MOESM1_ESM.pdf]

WELKOM

Voor wie is deze website?

Wat is het doel van deze website?

Hoe deze website te gebruiken?

Welkom op de website 'Beslissen over reumamedicatie'.

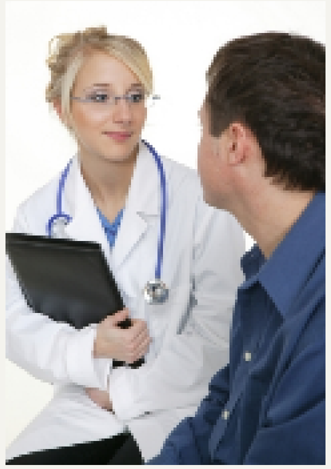

Reuma is een chronische aandoening. De behandeling van reuma wordt vaak bijgesteld en er worden regelmatig beslissingen genomen. Beslissingen over medicatie komen vaak voor. De reumatoloog beslist samen met u wat de mogelijkheden voor behandeling zijn. Deze website is een hulpmiddel om samen met uw reumatoloog een goed geïnformeerde beslissing te nemen over de medicijnen die u kunt gebruiken.

Deze website biedt informatie over reuma, de behandelopties en de medicijnen. Iedere behandeloptie heeft zijn voor- en nadelen en mogelijke risico's. De keuzehulp biedt u de mogelijkheid om uw persoonlijke voor- en nadelen af te wegen en uw vragen, wensen en zorgen duidelijk te krijgen, zodat u deze kunt meenemen naar het gesprek met de reumatoloog.

Voor wie is deze website bedoeld?

Deze website is bedoeld voor mensen met Reumatoïde Artritis, Artritis Psoriatica, en AnkyloSpondilitis (ziekte van Bechterew) die op het punt staan om een beslissing over reumamedicatie te maken.

Ook is deze website geschikt voor reumapatienten of hun familie/vrienden die zich willen verdiepen in de verschillende behandelopties.

Wat is het doel van deze website?

Het doel van deze website en met name de keuzehulp is om u te steunen om samen met uw reumatoloog een goed geïnformeerde beslissing te nemen over de medicijnen die u kunt gebruiken.

Hoe deze website te gebruiken?

Deze website biedt in eerste instantie een overzicht van alle behandelingsmogelijkheden. Vervolgens kunt u het proces van de keuzehulp doorlopen. U verkrijgt inzicht in zowel de verschillende behandelingsmogelijkheden als voor– en nadelen en mogelijke risico´s die met de behandelingen gepaard gaan.
De keuzehulp zal u niet aangeven welk medicijn in uw situatie het meest geschikt is, maar biedt ruimte om met uw persoonlijke voorkeuren en levenssituatie rekening te houden.

MOGELIJKE BEHANDELINGEN

Behandelaars

Medicijnen

Alternatieve behandeling

Kuren

Operaties

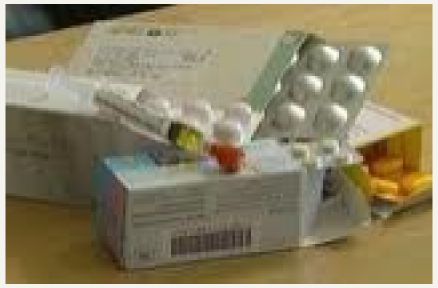

Mogelijke Behandelingen

Wat is voor u de beste behandeling nu u reuma heeft? Wie kan u helpen bij uw problemen? Welke medicijnen zijn er en waarvoor? Helpt een alternatieve behandeling? Heeft een kuur zin? Of een operatie? Allemaal vragen waarmee u te maken krijgt.

Behandelaars

Als u reuma heeft, krijgt u met verschillende behandelaars te maken. Behalve de **reumatoloog**, krijgt u bijvoorbeeld ook te maken met een **reumaverpleegkundige**, een **fysiotherapeut** en een **ergotherapeut**. Zij kunnen u helpen om te gaan met de gevolgen van reuma. *Meer informatie*

Medicijnen bij reuma

Reumatische aandoeningen zijn helaas nog niet te genezen. Er zijn wel medicijnen die de ontstekingen remmen of uw pijnklachten verminderen of die beide effecten hebben. Snel starten met medicijnen kan gewrichtsschade voorkomen. *Meer informatie*

Alternatieve behandeling

Misschien zoekt u ook naar alternatieve behandelingen. Sommigen merken dat zij hierbij baat hebben. Wilt u een alternatieve behandeling starten? Licht dan uw specialist hierover in. *Meer informatie*

Kuren

Kuren kunnen een goede aanvulling vormen op de behandeling die u al krijgt voor uw reuma. Na een kuur voelt u zich vaak beter. *Meer informatie*

Operaties

Soms is een operatie de enige uitweg om ernstige reumaklachten te behandelen. Dit hangt wel sterk af van uw persoonlijke situatie. Bij verschillende gewrichten zijn operaties mogelijk. Bijvoorbeeld aan schouder, elleboog, heup, knie en voet. Adviseert uw arts een operatie? Informeer dan uitgebreid naar de mogelijkheden en beperkingen van operaties. *Meer informatie*

INLEIDING

STAP 1: INFORMATIE VERZAMELEN

STAP 2: UW KEUZE

Inleiding - De medicatiekeuzehulp

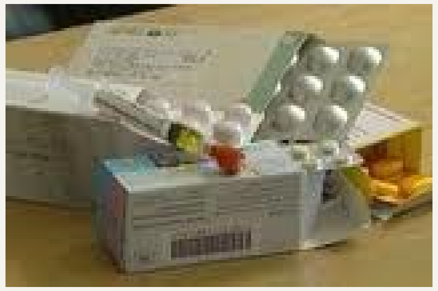

Het doel van deze keuzehulp is om u te steunen om samen met uw reumatoloog een goed geïnformeerde beslissing te nemen over de medicijnen die u kunt innemen.

Deze keuzehulp biedt informatie over reuma, de behandelopties en de medicijnen. Iedere behandeloptie heeft zijn voor- en nadelen en mogelijke risico's.

De keuzehulp zal u niet aangeven welk medicijn in uw situatie het meest geschikt is*. De keuzehulp werkt op basis van het advies van uw reumatoloog. Vervolgens biedt de keuzehulp u de mogelijkheid om uw persoonlijke voor- en nadelen af te wegen en uw vragen, wensen en zorgen duidelijk te krijgen, zodat u deze kunt meenemen naar het gesprek met de reumatoloog.

De keuzehulp bestaat uit enkele stappen:

STAP 1: INFORMATIE VERZAMELEN

STAP 2: UW KEUZE

* aan de uitkomst van de keuzehulp kunnen geen rechten worden ontleend.

Welke medicatie bestaat er en hoe werken deze?

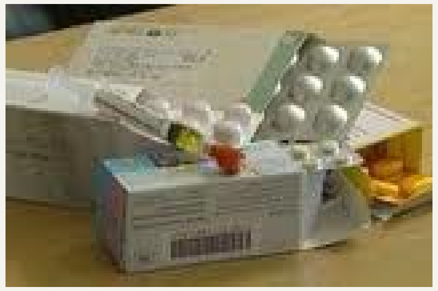

Medicijnen bij reuma
Reumatische aandoeningen zijn helaas nog niet te genezen.
Tegenwoordig schrijven reumatologen vaak al vroeg medicijnen voor, om schade aan uw gewrichten of organen zoveel mogelijk te verminderen. Artsen schrijven u verder medicijnen voor om de pijn en stijfheid te verminderen en de ontstekingen af te remmen.

Er zijn 4 categorieen medicijnen:
a. Eenvoudige pijnstillers
b. Ontstekingsremmende pijnstillers
c. Klasieke reumaremmers
d. Biologische reumaremmers

Eenvoudige pijnstillers
Lorem ipsum dolor sit amet, consectetur adipiscing elit. Quisque ante mi, molestie eget feugiat at, pharetra nec tellus. Nunc pellentesque massa placerat dolor pretium ut cursus arcu mollis. Praesent ac sem ipsum, congue elementum dolor. Aenean a varius sem. In a velit a eros interdum ullamcorper et at velit. Lorem ipsum dolor sit amet, consectetur adipiscing elit. Nunc posuere nunc id quam viverra pretium.

Ontstekingsremmende pijnstillers
Lorem ipsum dolor sit amet, consectetur adipiscing elit. Quisque ante mi, molestie eget feugiat at, pharetra nec tellus. Nunc pellentesque massa placerat dolor pretium ut cursus arcu mollis. Praesent ac sem ipsum, congue elementum dolor. Aenean a varius sem. In a velit a eros interdum ullamcorper et at velit.

Klassieke reumaremmers (DMARD's)
Lorem ipsum dolor sit amet, consectetur adipiscing elit. Quisque ante mi, molestie eget feugiat at, pharetra nec tellus. Nunc pellentesque massa placerat dolor pretium ut cursus arcu mollis. Praesent ac sem ipsum, congue elementum dolor. Aenean a varius sem. In a velit a eros interdum ullamcorper et at velit. Lorem ipsum dolor sit amet, consectetur adipiscing elit. Nunc posuere nunc id quam viverra pretium. Proin et turpis metus, sit amet sodales leo. Sed eget rhoncus libero. Vestibulum dignissim, eros viverra imperdiet pharetra, tortor leo adipiscing mi, non auctor orci elit at velit. Integer ligula libero, pretium in suscipit nec, feugiat quis justo.

Biologische reumaremmers
Lorem ipsum dolor sit amet, consectetur adipiscing elit. Quisque ante mi, molestie eget feugiat at, pharetra nec tellus. Nunc pellentesque massa placerat dolor pretium ut cursus arcu mollis. Praesent ac sem ipsum, congue elementum dolor. Aenean a varius sem. In a velit a eros interdum ullamcorper et at velit. Lorem ipsum dolor sit amet, consectetur adipiscing elit. Nunc posuere nunc id quam viverra pretium. Proin et turpis metus, sit amet sodales leo. Sed eget rhoncus libero.

>> >> GA DOOR NAAR STAP 1B

Beslissen over reumamedicatie

Home | Wat is Reuma? | Wat zijn behandelopties? | **Medicatie Keuzehulp**

INLEIDING

STAP 1: INFORMATIE VERZAMELEN

1A: Welke medicatie bestaat er en hoe werken deze?

1B: Het advies van uw reumatoloog.

1C: Medicatie vergelijken - Voordelen, nadelen en risico's op een rij.

STAP 2: UW KEUZE

Het advies van uw reumatoloog

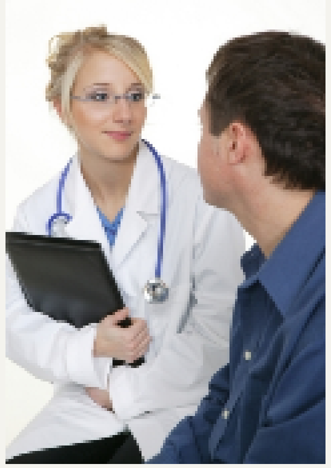

Uw reumatoloog heeft u een persoonlijk advies gegeven over mogelijk geschikte medicatie voor u.
Ter aanvulling op dit advies kunt u op deze website de verschillende medicijnen uit dit advies met elkaar vergelijken.

Welk advies heeft uw reumatoloog u gegeven? Kies er 1 en klik op *verder*

- [ ] starten met een klassieke reumaremmer of een combinatie van klassieke reumaremmers
- [ ] starten met een biologische reumaremmer*
- [x] starten met een biologische reumaremmer gecombineerd met een klassieke reumaremmer*

** Biologische reumaremmers kunnen alleen worden voorgeschreven aan reumapatiënten bij wie de klassieke behandeling tweemaal gefaald heeft of die de klassieke behandeling niet kunnen verdragen.*

VERDER

Over welke specifieke medicatie hebt u met uw reumatoloog gesproken?

Klassieke reumaremmers

- [x] Reumarem
- [x] Antireuma
- [ ] Stopreuma

Biologische reumaremmers

- [x] Bioreuma*
- [ ] Artrobio

>> >> GA DOOR NAAR STAP 1C EN VERGELIJK

>> >> SLA DEZE STAP OVER EN GA NAAR STAP 2: UW KEUZE

INLEIDING

STAP 1: INFORMATIE VERZAMELEN

1A: Welke medicatie bestaat er en hoe werken deze?

1B: Het advies van uw reumatoloog.

1C: Medicatie vergelijken - Voordelen, nadelen en risico's op een rij.

STAP 2: UW KEUZE

Bioreuma | Reumarem | Anti-Reuma

Bioreuma | Reumarem | Anti-Reuma

Overige medicatie...

Klik op de buttons om de kaarten te bekijken

Klik op de buttons om de kaarten te bekijken

>> >> GA DOOR NAAR STAP 2: UW KEUS

INLEIDING

STAP 1: INFORMATIE VERZAMELEN

1A: Welke medicatie bestaat er en hoe werken deze?

1B: Het advies van uw reumatoloog.

1C: Medicatie vergelijken - Voordelen, nadelen en risico's op een rij.

STAP 2: UW KEUZE

Bioreuma | Reumarem | Anti-Reuma | Bioreuma | Reumarem | Anti-Reuma

	Bioreuma	Anti-Reuma
**Effect**	bij 65-70% van de gebruikers is er een lichte tot grote verbetering in het aantal gevoelige en gezwollen gewrichten. Effect voelbaar na 1-3 maanden. meer informatie	bij 55-62% van de gebruikers is er een lichte tot grote verbetering in het aantal gevoelige en gezwollen gewrichten. Effect voelbaar na 1 maand. meer informatie
**Bijwerkingen en risico's**	**Veelvoorkomend:** De eerste dagen na het infuus kunnen patienten zich misselijk voelen. Daarnaast kan het van invloed zijn op uw eetlust; u hebt meer behoefte aan eten. **Zeldzaam:** Het is mogelijk dat u neerslachtig of depressief wordt van Bioreuma. meer informatie	**Veelvoorkomend:** Men kan blauwe plekken krijgen van het injecteren. **Zeldzaam:** Het is mogelijk dat u neerslachtig of depressief wordt van Antireuma. meer informatie
**Dagelijks gebruik en bewaren**	1x per 2 week infuus in ziekenhuis Wordt in ziekenhuis bewaard. meer informatie 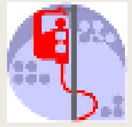	1x per week injecteren. Deze medicatie dient in de koelkast bewaard te worden. meer informatie 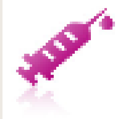
**Controles / Bloedwaarden testen**	Een keer per 3 maand vinden er bloedcontroles plaats om o.a. de leverwaarden te controleren. meer informatie	Een keer per 3 maand vinden er bloedcontroles plaats om o.a. de leverwaarden te controleren. meer informatie
**Wanneer niet gebruiken?**	Bioreuma mag niet gebruikt worden wanneer u bekend bent met nierfalen. meer informatie	Antireuma mag niet gebruikt worden wanneer u bekend bent met nierfalen. meer informatie
**Interacties met andere medicijnen**	Bioreuma mag niet gebruikt worden in combinatie met bloedverdunners. meer informatie	Antireuma mag niet gebruikt worden in combinatie met bloedverdunners. meer informatie
**Gevolgen voor dagelijks leven en ervaringsverhalen**	Toediening van Bioreuma vind plaats in het ziekenhuis en neemt 1 dagdeel per 2 weken in beslag. Dit kan van invloed zijn op uw taken en rollen (denk aan werk, verzorging van kinderen, etc).<br><br>Kijk en luister naar de ervaringen van Anouschka, Cecile, Edwin en Paul met Bioreuma: *Klik op de foto om het filmpje te starten* 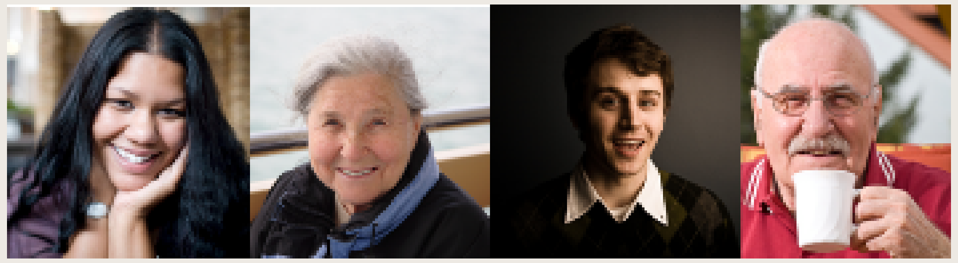	Antireuma wordt per injectie toegediend. Veel mensen zien er tegenop om te moeten injecteren. U krijgt de eerst uitgebreid instructie. Het is ook mogelijk om het injecteren bij de huisarts-assistente te laten doen. Ook is het mogelijk dat iemand uit uw omgeving geinstrueerd wordt.<br><br>Kijk en luister naar de ervaringen van Fatima, Hans, Teun en Gigi met Bioreuma: *Klik op de foto om het filmpje te starten* 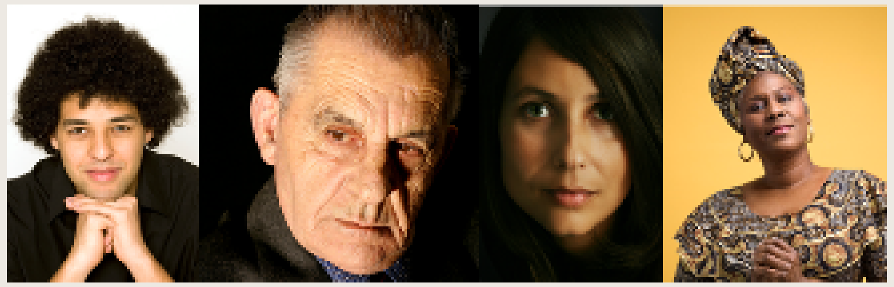

>> >> GA DOOR NAAR STAP 2: UW KEUS

Wat vind u belangrijk?

Nu u weet welke mogelijkheden er zijn, kunt u gaan nadenken over wat belangrijk is voor u persoonlijk. De vragen in dit scherm helpen u om vervolgens samen met de reumatoloog de behandeling te kiezen die het beste bij u past.

Hoe belangrijk is het voor u dat het medicijn snel effect heeft?

- ☐ Heel belangrijk
- ☐ Een beetje belangrijk
- ☐ Niet belangrijk

Als u zou beslissen om met deze medicatie te starten, hoe zou u zich dan voelen over de mogelijke bijwerkingen?

- ☐ Ik zou me erg angstig voelen.
- ☐ Ik zou me een beetje zorgen maken.
- ☐ Ik zou me geen zorgen maken.

Hoe belemmerend is het voor u om eens per week naar het ziekenhuis te komen?

- ☐ Geen probleem
- ☐ Een beetje een probleem
- ☐ Groot probleem

Past het in uw leven om medicatie koel te moeten bewaren?

- ☐ Geen probleem
- ☐ Een beetje een probleem
- ☐ Groot probleem

In hoeverre bent u bereid om regelmatig uw bloed te laten testen (voor nier- of leverfalen)?

- ☐ Geen probleem voor mij
- ☐ Een beetje een probleem voor mij
- ☐ Groot probleem voor mij

Hoe belemmerend is het voor u om eens per week een injectie toe te dienen (evt met hulp)?

- ☐ Geen probleem
- ☐ Een beetje een probleem
- ☐ Groot probleem

>> >> Verder

INLEIDING
STAP 1: INFORMATIE VERZAMELEN
STAP 2: UW KEUZE

Wat vind u belangrijk?

Wanneer u praat met uw reumatoloog is het van belang dat u verteld wat voor u belangrijk is. Zo kan de reumatoloog daarmee rekening houden in zijn advies.

Hieronder staan enkele voorbeelden van veelvoorkomende vragen. Kruis aan welke voor u van belang zijn en vul eventueel aan.

vragen

- ☐ Ik gebruik ook andere medicatie. Gaat dat samen?
- ☐ Hoe vaak moet ik voor bloedcontroles komen?
- ☐ Voeg zelf een vraag toe...

Zorgen

- ☐ Ik vind het spannend om te gaan spuiten. Kan ik hier begeleiding in krijgen?
- ☐ Hoe groot is de kans voor mij om leveraandoening te krijgen?
- ☐ Voeg zelf een vraag toe...

Voorkeuren

- ☐ Ik wil graag flexibel zijn en zelf de behandeling kunnen toepassen.
- ☐ Ik wil graag dat mijn partner ook instructie krijgt.
- ☐ Voeg zelf een voorkeur toe...

Heeft u al een voorkeur voor een medicijn?

- ☐ Nee, ik heb nog geen voorkeur.
- ☐ Ja, ik heb een voorkeur, namelijk: Kies hier uw medicijn

Ruimte voor toelichting en notities

>> >> Verder

Samenvatting van uw resultaten

Nu u weet wat uw opties zijn en inzicht heeft in uw opvattingen en zorgen hierover, kunt u gaan praten met uw dokter, familie en vrienden.
Op deze pagina vind u een samenvatting van uw antwoorden. Deze kunt u ook downloaden. Ook kunt u uw antwoorden emailen. Dit formulier kunt u meenemen naar het gesprek (met uw reumatoloog) en gebruiken als steuntje in de rug.

DOWNLOAD ☐ Email uw antwoorden: Vul hier uw emailadres in

Wat vind ik belangrijk?

Ik vind het belangrijk dat het medicijn snel effect heeft
Ik vind het lastig als ik steeds naar het ziekenhuis moet.

Mijn Vragen, Zorgen en Twijfels
Ik maak me zorgen over de mogelijke bijwerkingen.
Wat zijn mijn persoonlijke kansen?
Welke alternatieven zijn nog meer geschikt voor mij?
De medicatie dient in de koelkast bewaard te worden. Hoe doe ik dat tijdens mijn vakantie?

Mijn voorkeuren
Ik wil graag flexibel zijn.

Momenteel gaat mijn voorkeur uit naar Bioreuma.
Notities en Toelichtingen: Mijn tweede voorkeur gaat uit naar Reumarem
