# Supplementary material for: Development of a web-based patient decision aid for initiating disease modifying anti-rheumatic drugs using user-centred design methods
Source: BMC Med Inform Decis Mak. 2017 Apr 26;17:51. doi: 10.1186/s12911-017-0433-5 (PMC5405550; doi:10.1186/s12911-017-0433-5)
Supplement: Supplementary file 3 — Design process from paper prototype to working prototype and final version. Description: Screenshots of the page that allows patients to compare DMARDs, illustrating the design process from paper prototype to working prototype and final version. (DOCX 573 kb) [file 12911_2017_433_MOESM2_ESM.docx]

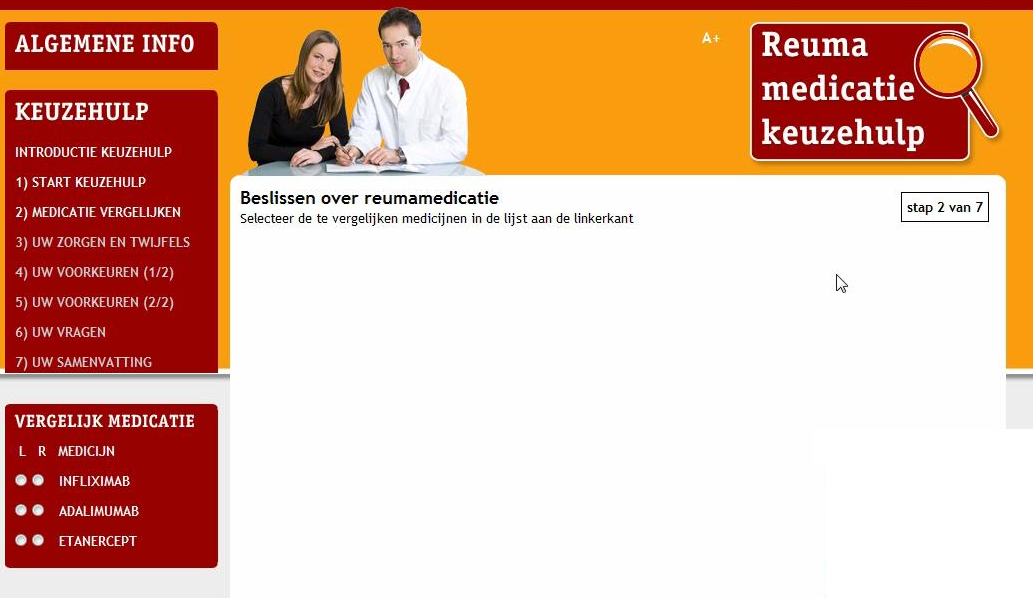
Screenshot 1: First draft (paper prototype) of
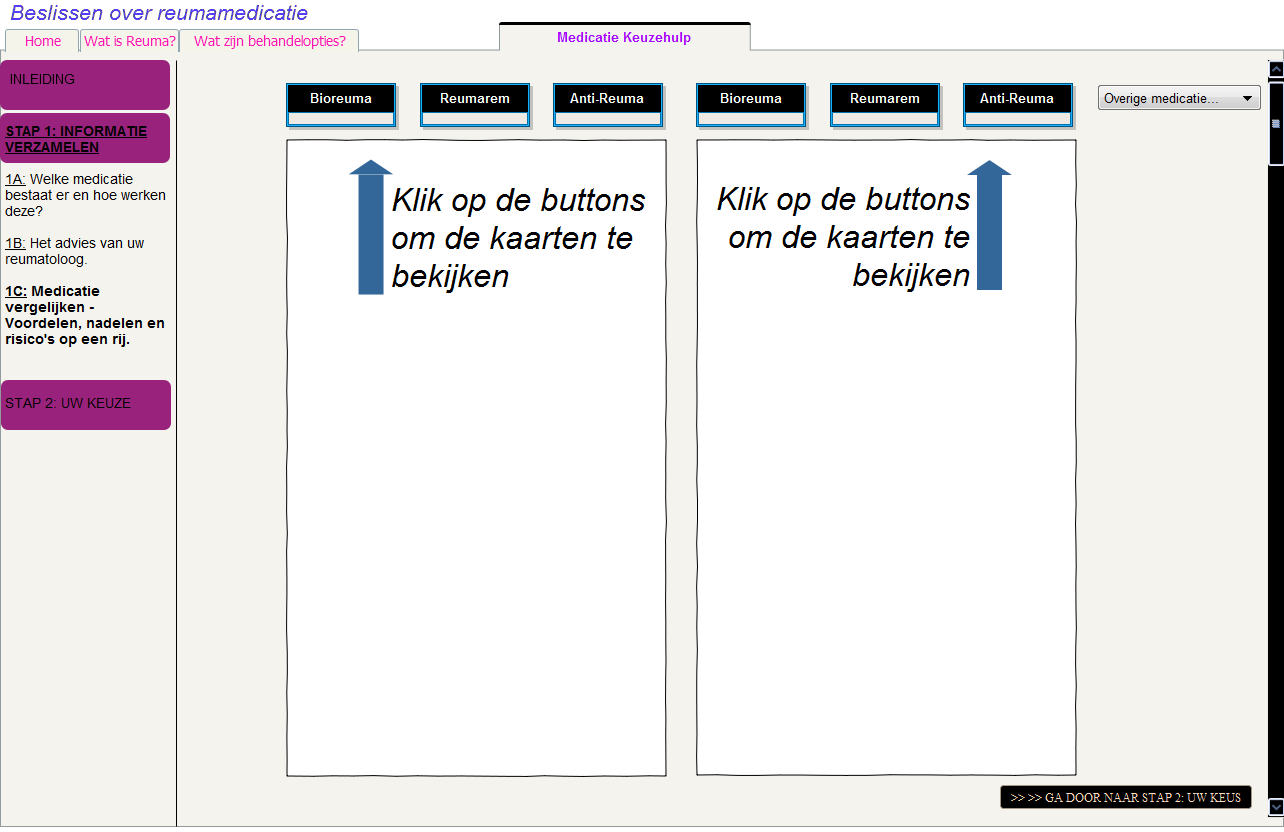
the screen used during the needs assessment. This screen enabled patients to compare medications. The fictional medication names “Bioreuma,” “Reumarum” and “Anti-Reuma” were used to avoid providing incorrect information to patients.

Screenshot 2: The working prototype of the screen used in the usability study. This screen enabled patients to compare medications. The study revealed that the placement of two medicines side-by-side was not intuitive or easy to use. (See the left-hand corner box titled “Vergelijk Medicatie” with the buttons L, R and Medicijn.)


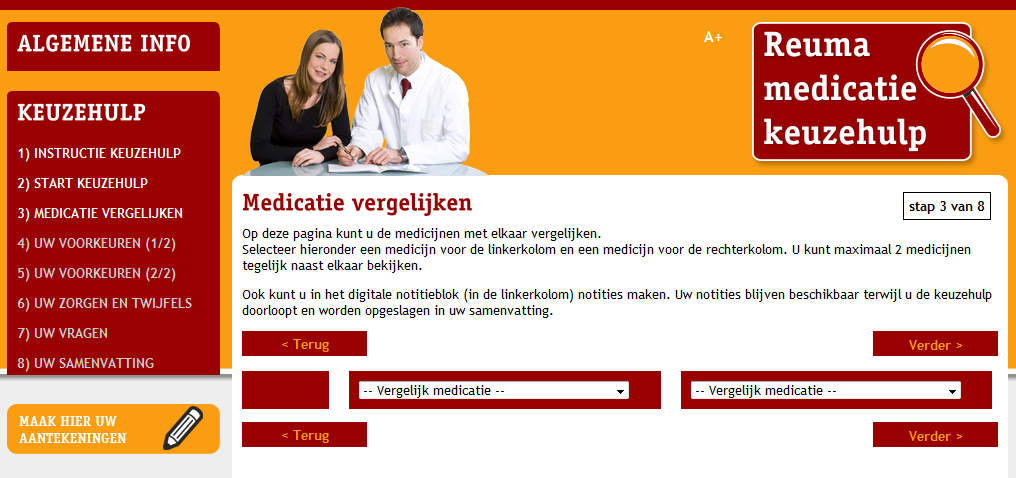
Screenshot 3: Final version of the screen that enabled patients to compare medications. User navigation was adjusted based on results of the usability study.
