# Supplementary figures and images for: Development of a web-based patient decision aid for initiating disease modifying anti-rheumatic drugs using user-centred design methods
Source: BMC Med Inform Decis Mak. 2017 Apr 26;17:51. doi: 10.1186/s12911-017-0433-5 (PMC5405550; doi:10.1186/s12911-017-0433-5)

**
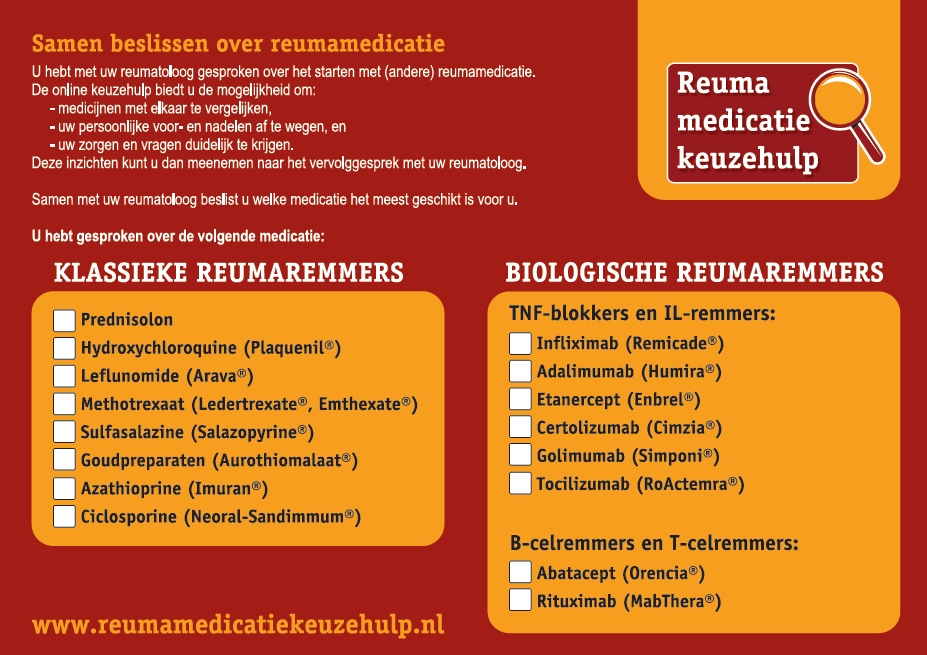
**

Supplement: Supplementary file 4 — Card to refer to the Patient Decision Aid. Description: Image of the card rheumatologists use to refer patients to the Patient Decision Aid. (DOCX 139 kb) [file 12911_2017_433_MOESM3_ESM.docx]
